# Novel ATP-Independent RNA Annealing Activity of the Dengue Virus NS3 Helicase

**DOI:** 10.1371/journal.pone.0036244

**Published:** 2012-04-30

**Authors:** Leopoldo G. Gebhard, Sergio B. Kaufman, Andrea V. Gamarnik

**Affiliations:** 1 Fundación Instituto Leloir-Consejo Nacional de Investigaciones Científicas y Técnicas, Ciudad de Buenos Aires, Argentina; 2 Instituto de Química y Fisicoquímica Biológicas y Departamento de Química Biológica, Facultad de Farmacia y Bioquímica, Universidad de Buenos Aires, Ciudad de Buenos Aires, Argentina; International Centre for Genetic Engineering and Biotechnology, Italy

## Abstract

The flavivirus nonstructural protein 3 (NS3) bears multiple enzymatic activities and represents an attractive target for antiviral intervention. NS3 contains the viral serine protease at the N-terminus and ATPase, RTPase, and helicase activities at the C-terminus. These activities are essential for viral replication; however, the biological role of RNA remodeling by NS3 helicase during the viral life cycle is still unclear. Secondary and tertiary RNA structures present in the viral genome are crucial for viral replication. Here, we used the NS3 protein from dengue virus to investigate functions of NS3 associated to changes in RNA structures. Using different NS3 variants, we characterized a domain spanning residues 171 to 618 that displays ATPase and RNA unwinding activities similar to those observed for the full-length protein. Interestingly, we found that, besides the RNA unwinding activity, dengue virus NS3 greatly accelerates annealing of complementary RNA strands with viral or non-viral sequences. This new activity was found to be ATP-independent. It was determined that a mutated NS3 lacking ATPase activity retained full-RNA annealing activity. Using an ATP regeneration system and different ATP concentrations, we observed that NS3 establishes an ATP-dependent steady state between RNA unwinding and annealing, allowing modulation of the two opposing activities of this enzyme through ATP concentration. In addition, we observed that NS3 enhanced RNA-RNA interactions between molecules representing the ends of the viral genome that are known to be necessary for viral RNA synthesis. We propose that, according to the ATP availability, NS3 could function regulating the folding or unfolding of viral RNA structures.

## Introduction

Proteins with RNA helicase activity have been identified in the genome of plus stranded RNA viruses [1], [2]. It has been proposed that these enzymes facilitate the activity of RNA-dependent RNA-polymerases during genome amplification by unwinding secondary and tertiary RNA structures, or separating RNA duplexes including viral plus and minus strands. Viral helicases may also serve as RNPases, stripping proteins from the viral RNA or remodelling RNA structures that may function as modulators of the viral processes [3]–[5]. Viral RNA genomes contain a wide variety of RNA regulatory signals in the coding and non-coding regions. These signals function as promoters, enhancers, and repressors of translation, transcription, replication, and encapsidation [6]–[17]. Therefore, the participation of chaperones and helicases regulating viral RNA structures likely modulate RNA function. Although it is widely accepted that viral helicases are key components of the viral replication machinery, the mechanisms by which these enzymes participate during the replication process are still unclear. Here, we investigated different activities associated to the dengue virus (DENV) RNA helicase encoded in the NS3 protein.

DENV is a member of the *Flavivirus* genus in the *Flaviviridae* family, together with other important human pathogens such as Yellow Fever virus (YFV), West Nile virus (WNV), Saint Louis encephalitis virus (SLEV), and Japanese encephalitis virus (JEV) [18]. DENV is the most significant mosquito borne human viral pathogen worldwide, and is responsible for the highest rates of disease and mortality among the members of the *Flavivirus* genus [19]. The viral genome is a single-stranded RNA molecule of positive polarity of about 11 kb in length, which encodes a long polyprotein that is co- and post-translationally processed by host and viral proteases to yield three structural proteins (C, prM, and E), and at least seven non-structural proteins (NS1, NS2A, NS2B, NS3, NS4A, NS4B, and NS5) [20].

NS3 is a multifunctional protein essential for flavivirus replication [21], [22]. The N-terminal third of this polypeptide forms a two-component serine protease domain together with its cofactor NS2B [23]–[26]. This viral protease is involved in processing the viral polyprotein. The minimal region retaining protease activity of DENV2 NS3 was mapped to the N-terminal 167 amino acids, and requires the central 40 amino acid region of the membrane-associated NS2B cofactor [27]. The C-terminus of NS3 has been first predicted and then demonstrated to have three different enzymatic activities: NTPase, RNA triphosphatase (RTPase), and helicase [2], [27]–[29]. The RTPase activity that cleaves the phosphoric anhydride bond of 5′-triphosphorylated RNA, is the first of three sequential enzymatic reactions of RNA 5′ capping, which is essential for viral translation and RNA stability (for review see [30]). The RNA unwinding activity of DENV NS3 was first reported by Padmanabhan and collaborators [27]. Mutational analysis has indicated that the ATPase/helicase and RTPase activities of DENV NS3 share a common active site [28], [31]. Crystal structures for the active NS3 protease domain [32], [33], the helicase domain [34]–[37], and the full length NS3 molecule [38] have been reported. In addition, important information was provided from crystal structures of DENV helicase in complex with single-stranded RNA and different ATP analogues [39]. These structural studies indicated that RNA binding induces a conformational change in the NS3 to a closed form, while no changes in protein structure were observed in different nucleotide-bound states.

The helicase activity of hepatitis C virus (HCV) NS3, member of the *Hepacivirus* genus in the *Flaviviridae* family, has been extensively studied [40]–[46]. In contrast, less is known about mechanistic aspects of helicases from mosquito borne flaviviruses. A recent study has reported interesting biochemical properties of the DENV helicase [47]. It was found that the enzyme preferentially binds single-stranded RNA, while low affinity was observed for single or double-stranded DNA (dsDNA) molecules. In addition, it was shown that DENV NS3 unwinds RNA duplexes with a 3′ to 5′ directionality, moving along a tracking RNA strand. In contrast to the NS3 from HCV, the DENV enzyme displays low processivity, unwinds dsDNA molecules inefficiently, and exhibits an RNA triphosphatase activity [47]. Comparison of crystal structures of the full-length NS3 from DENV and HCV indicated a major difference in the relative orientations between the protease and helicase domains in the two proteins. Specifically, a beta-strand in the HCV NS3 clamps the protease domain next to the helicase domain, thereby creating a compact conformational state that differs from the extended conformation observed in the DENV protein [38], [40].

In this study, we investigated enzymatic properties of the DENV NS3 protein in regard to its ability to modulate the formation of RNA structures. Using in vitro assays to evaluate RNA unwinding and annealing reactions, we identified a new RNA strand annealing activity of NS3. In contrast to the requirements for the helicase activity, we demonstrated that the RNA annealing activity does not require ATP. The viral NS3 establishes an ATP-dependent steady state between RNA unwinding and annealing, allowing modulation of the two opposing activities of this enzyme through ATP concentration. Our findings provide novel information for functional roles of the DENV NS3 in regulating viral RNA structures.

## Results

### Dengue virus full-length NS3 and the helicase domain show similar ATPase and RNA unwinding activities

**Figure 1:**
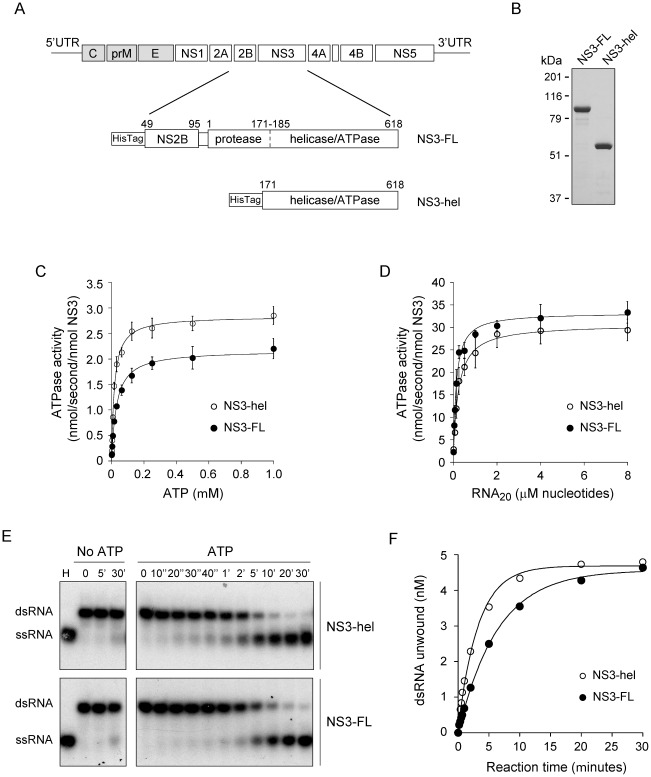
Enzymatic activities of NS3 full-length and the helicase domain. (A) Schematic diagram of DENV genome organization and recombinant NS3-FL and NS3-hel proteins. NS3-FL includes the substitution His51Ala in the protease domain to avoid protein degradation. The construct also contains 47 residues of the cofactor NS2B, as indicated in the scheme. (B) Representative gel showing expression and purification of the two variants of NS3. (C) ATPase activity of NS3-FL (filled circles) and NS3-hel (open circles). The initial velocities of ATP hydrolysis were determined at 25°C in the presence of the indicated concentrations of ATP. Data points represent the mean of three independent determinations and error bars indicate +/− one standard deviation. The continuous lines are the plots of Michaelis-Menten for the best fitting of *K*_M_ and *k*_cat_. (D) RNA-stimulated ATPase activities of NS3-FL and NS3-hel. The initial velocities of ATP hydrolysis were determined at 25°C in the presence of 2.0 mM ATP-MgCl_2_ and the indicated concentrations of RNA (20-mer). The initial velocities of ATP hydrolysis were fitted to a hyperbolic equation by nonlinear regression analysis. Data points represent the mean of three independent determinations and error bars indicate +/− one standard deviation. (E) Representative gel of RNA unwinding time courses of NS3-FL and NS3-hel. Unwinding reactions were carried out at 30°C with 250 nM of enzyme and 5 nM of dsRNA substrate (see Materials and Methods). Reactions were initiated by addition of 2.0 mM ATP-MgCl_2_ together with 100 nM RNA trap, and terminated at the indicated times (as indicated on top of the gel). Time 0 represents the unwinding reactions before ATP addition. Lane H represents a positive control (heat denatured duplex). Negative controls (No ATP) were included, as indicated on top of the gel. Samples were resolved by polyacrilamide gel electrophoresis in non-denaturing conditions. Mobility of double-stranded (dsRNA) and single-stranded RNA (ssRNA) is indicated on the left. (F) Representative time courses of RNA unwinding reactions obtained by quantification of bands from the gel shown in E. The continuous lines represent the best fits to a mono-exponential equation. Initial velocity for unwinding reactions were obtained as described in Materials and Methods.

The flavivirus NS3 protein contains two defined domains with distinct enzymatic activities [27]. Although the helicase domain, comprising amino acids 171 to 618, is an active enzyme, the protease domain has been proposed to have a modulatory role on the helicase and NTPase activities [35], [37], [38]. To further analyze this possibility, two recombinant variants of DENV NS3 were obtained (Fig. 1A and 1B). The first variant represented a single polypeptide that mimics the NS2B–NS3 complex containing the full-length NS3 (618 amino acids) linked to the NS2B hydrophilic region (47 amino acids) as previously described [48]. This variant named NS3-FL, also carried a mutation in the protease catalytic site (H51A) to avoid autoproteolysis [48]. The second variant represented a truncated NS3, containing the helicase domain (amino acids 171 to 618) (Fig. 1A).

The recombinant proteins were used to determine comparatively ATPase and RNA unwinding activities. Regarding ATP hydrolysis, the two variants (NS3-FL and NS3-hel) showed hyperbolic kinetics as a function of substrate concentration, with catalytic constants (*k*_cat_) of 2.2±0.1 s^−1^ and 2.8±0.1 s^−1^; and Michaelis Menten constants (*K*_M_) of 31±3 µM and 17±2 µM, respectively (Fig. 1C). The results indicate that the turnover value and affinity for ATP were similar for the two variants. It has been previously demonstrated that viral ATPases, including NS3 from flaviviruses, are stimulated by single-stranded RNA [27], [29], [49]–[53]. Structural studies have previously shown major conformational changes in NS3 upon RNA binding, supporting the idea that allosteric interactions between the RNA binding site and the ATPase catalytic site exist [39]. To test whether NS3-FL and NS3-hel preserved this property, we measured the RNA stimulated ATPase activities. At RNA saturation, we observed an increase of 13.5-fold and 9.6-fold with respect to the basal activities for NS3-FL and NS3-hel, respectively (Fig. 1D).

To study the RNA helicase activity of the two variants, ^32^P- or Cy5-labeled double-stranded RNA substrates were used. This substrate consisted of a labeled 30 nucleotide-long RNA strand hybridized to a second 15 nucleotide-long complementary RNA, leaving at the 3′ end a 15 nucleotide-long single-stranded overhang. The unwinding activity was evaluated by electrophoretic mobility shift assays in native polyacrylamide gels by detecting the labeled RNA strand as single or double-stranded species. For the assay, both enzymes were pre-incubated 10 min with the RNA duplex, and then the unwinding reaction was started by adding ATP. In a reaction mix containing the same components but in the absence of ATP, the substrate remains mainly as double-stranded (Fig. 1E, left panels). In the presence of ATP, the unwinding time courses for both enzymes show similar behavior with mono-exponential kinetics with comparable amplitudes (*A* ^unw^) of 4.57±0.05 nM and 4.54±0.11 nM, and apparent RNA unwinding rate constants of 0.16±0.01 min^−1^ and 0.31±0.02 min^−1^, for NS3-FL and NS3-hel, respectively (Fig. 1E and 1F).

**Figure 2:**
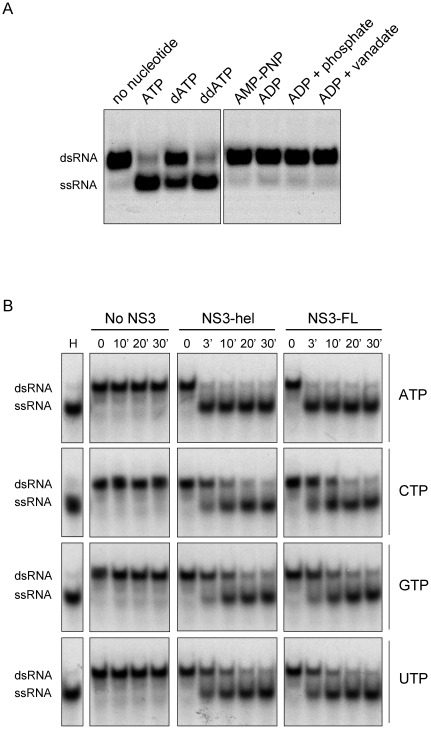
NTP utilization in RNA unwinding reactions. (A) Different nucleotides were tested as substrates for the RNA unwinding activity of NS3-hel. Unwinding reactions were carried out at 30°C for 30 minutes as described in Fig. 1E including 1.0 mM of the respective nucleotide as indicated on top of each lane. A negative control (no nucleotide) was included. (B) Usage of canonical NTPs for the RNA unwinding activity of NS3-FL and NS3-hel. Unwinding reactions were carried out as described in Fig. 1E, but incubated at 37°C in the presence of 2.0 mM of ATP, CTP, GTP or UTP as indicated on the right. Lane H represents a positive control (heat denatured duplex). Negative control (no NS3), NS3-hel, or NS3-FL were included as indicated on top of the gel. Mobility of double-stranded (dsRNA) and single-stranded RNA probe (ssRNA) is indicated on the left.

It has been reported for different RNA helicases that not only NTPs but also dNTPs and other nucleotide analogues are able to drive the RNA unwinding activity [54], [55]. To evaluate the ability of the DENV NS3 helicase to use different substrates as energy source, the unwinding reaction was started by adding different NTP analogues (Fig. 2A). Virtually no helicase activity was observed in the presence of the non-hydrolyzable ATP analogue AMP-PNP or in the presence of ADP. In addition, the enzyme was inactive in the presence of the transition state analogue ADP plus orthovanadate (Fig. 2A). In contrast, both dATP and ddATP were suitable sources of energy, indicating the requirement of a substrate with hydrolyzable phosphodiester group at gamma position. To evaluate the ability of each NTP to support RNA unwinding by NS3-FL and NS3-hel, reactions were performed in the presence of ATP, CTP, GTP and UTP. The four NTPs were efficient substrates for RNA unwinding without apparent difference between the two proteins under the conditions tested (Fig. 2B). It is important to note that for both enzymes, the helicase unwinding rates with ATP were significantly faster than with the other nucleotides (Fig. 2B).

The results indicate that the ATPase and helicase activities of DENV NS3 are not significantly affected by the protease domain.

### NS3 establishes an ATP-dependent steady–state between RNA unwinding and strand annealing

**Figure 3:**
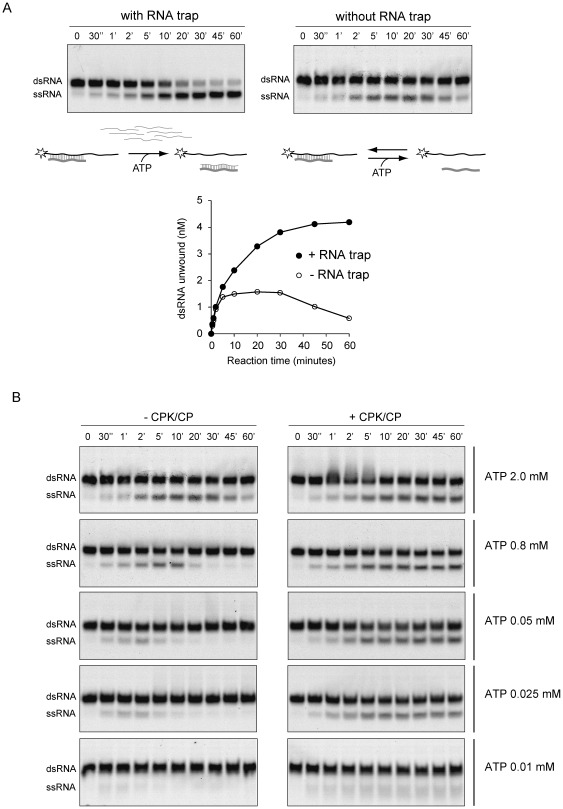
ATP-dependent steady-state RNA unwinding activity of NS3. (A) Effect of an RNA trap on NS3 unwinding activity. Representative gels showing time courses of unwinding reactions of NS3-hel in the presence (left panel) and absence (right panel) of an RNA trap at 20-fold molar excess respect to the probe. Underneath of each gel, a schematic representation of the reaction is shown. In the presence of RNA trap, the decoy RNA binds the unlabeled ssRNA and avoids re-annealing, allowing completion of the reaction. In the absence of RNA trap, both reactions, RNA unwinding and RNA annealing, are observed. (B) RNA unwinding activity of NS3 in the presence of an ATP regeneration system. Representative gels showing time courses of unwinding reactions of NS3-hel in the absence or presence of an ATP regeneration system (CPK/CP), left and right panels, respectively. Each reaction was carried out at the indicated initial ATP concentration (shown on the right). Mobility of double-stranded (dsRNA) and single-stranded RNA probe (ssRNA) is indicated on the left.

The standard RNA unwinding time course included an RNA trap to prevent annealing of the unwound substrate. This assay allows following RNA unwinding without detecting the reverse reaction. To further study the properties of DENV NS3, we analyzed RNA unwinding in the presence or absence of RNA trap (Fig. 3A). In the presence of a 20-fold molar excess of the decoy RNA respect to the probe, NS3 readily catalyzed duplex unwinding to almost completion (Fig. 3A, left panel). In the absence of RNA trap, the RNA unwinding time course showed three distinct phases (Fig. 3A, right panel and graph). The first phase is characterized by a superimposable curve respect to the one obtained in the presence of trap (Fig. 3A, graph). In this phase, NS3 unwinds up to 35% of the substrate. The second phase is characterized by a prolonged steady state (10 to 30 minutes). The last phase shows reversion of the reaction, in which the unwound RNA strands re-anneal. The reversibility of this reaction could be explained by ATP depletion. To study this possibility, we carried out experiments where NS3 was incubated in the presence and absence of an ATP regeneration system. Creatine phosphokinase (CPK) and creatine phosphate (CP) were included in the reaction media with different concentrations of ATP (0.01, 0.025, 0.05, 0.8, and 2 mM). In the presence of the ATP regeneration system, but in the absence of RNA trap, the reaction reached a steady state in about 5–10 minutes with amplitudes ranging from 5 to 35% (Fig. 3B, right panels). In the absence of the ATP regeneration system, re-annealing of the single- stranded molecules was readily observed and the amplitude of the reaction depended on the initial ATP concentration (Fig. 3B, left panels).

These results indicate that reversion of the unwinding reaction was due to the consumption of ATP. When ATP is not limiting the reaction, the third phase of the time course (in the absence of RNA trap) disappears. A steady state, indicating equal velocity between forward (RNA unwinding) and reverse (RNA annealing) reactions, is observed. However, it was uncertain whether the steady state reflected the equilibrium between the helicase-mediated RNA unwinding and the spontaneous RNA annealing, or if the latter reaction was also accelerated by NS3.

### DENV NS3 displays ATP-independent RNA annealing activity

**Figure 4:**
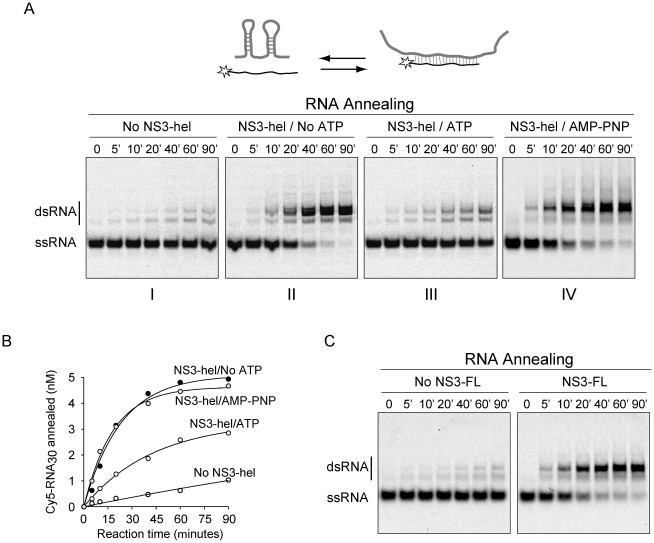
RNA strand annealing activity of NS3. (A) NS3-hel accelerates annealing of structured RNAs. The annealing reactions were started by mixing aliquots of the two RNAs as single-stranded species. On top, a schematic representation of the annealing process is shown. Representative gels of RNA annealing time courses in the absence and presence of NS3-hel, ATP, and AMP-PNP (as indicated on top of each gel). The substrate included a highly structured RNA molecule and a fluorescently labeled complementary RNA. Annealing reactions were incubated at the indicated times (top of each gel). (B) A graph showing representative time courses of RNA annealing obtained by quantification of bands in the gel. The continuous lines represent the best fits to the differential equations for a one-step bimolecular reaction (see Materials and Methods). (C) Representative gels of RNA annealing time courses in the absence and presence of NS3-FL. Reaction conditions, including substrate and protein concentrations, were identical as in (A).

To study whether NS3 modulates the annealing of complementary RNA strands, a similar assay used to determine RNA unwinding was applied to evaluate the reverse reaction. For this purpose, new RNAs were designed in such a way that spontaneous hybridization was kinetically unfavorable. We designed a structured 58 nucleotide-long RNA that was predicted to form two stable hairpins. This RNA was complementary to an unstructured 30 nucleotide-long Cy5-labeled RNA. As expected, spontaneous annealing of these two RNAs was slow with an initial velocity of 0.012±0.03 nM min^−1^ (Fig. 4A, panel I). Interestingly, the process of duplex formation was greatly accelerated by the presence of NS3-hel with an initial velocity of 0.277±0.025 nM min^−1^ (Fig. 4A, panel II). In identical conditions but in the presence of 0.2 mM ATP (with an ATP regeneration system), the initial velocity of RNA annealing was 0.095±0.004 nM min^−1^ (Fig. 4A, panel III). Full hybridization of the two RNA molecules was only observed in the absence of ATP (Fig. 4B). These results indicate that NS3 promotes RNA hybridization; however, ATP can function as a modulator of the unwinding/annealing reactions. To analyze whether binding of a non-hydrolyzable ATP analogue to NS3 modulates the RNA annealing activity, NS3-hel was incubated with AMP-PNP. In the presence of 0.2 mM AMP-PNP, the initial velocity of the reaction was 0.271±0.019 nM min^−1^ (Fig. 4A, panel IV). The results indicate that this ATP analogue did not modify significantly the RNA annealing activity of NS3, indicating that ATP hydrolysis, and not ATP binding, is necessary for RNA annealing modulation.

We have shown in Figure 1 and 2 similar helicase and NTPase activities for NS3-hel and NS3-FL. Here, we analyze whether NS3-FL also promotes RNA annealing. To this end, a similar assay as the one described in Figure 4A was used employing NS3-FL. RNA duplex formation was readily observed in the presence of protein, with an initial velocity of 0.308±0.015 nM min^−1^ (Fig. 4C), indicating that both NS3-hel and NS3-FL have similar RNA annealing activity.

**Figure 5:**
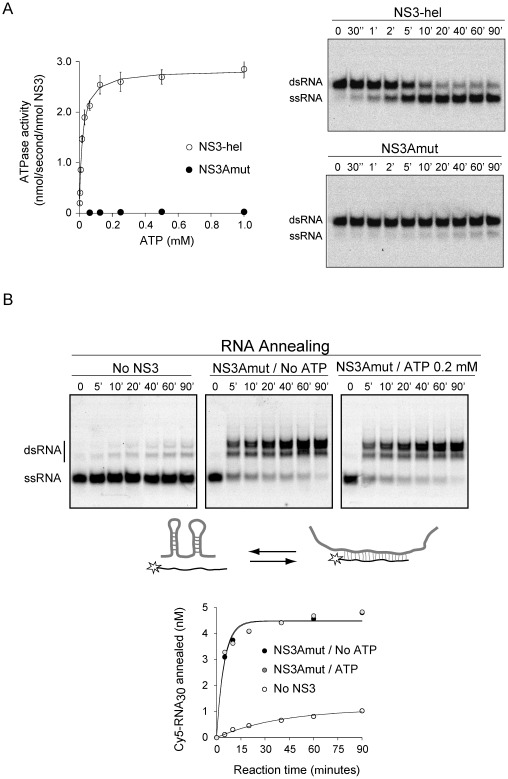
ATPase/helicase defective NS3-hel mutant (NS3Amut) conserved RNA annealing activity. (A) ATPase and RNA helicase activities of a variant of NS3 carrying a two-amino acid substitution (D284A-E285A). On the left, ATP hydrolysis time course for WT and mutant NS3. On the right, representative gels of RNA unwinding time courses for WT and mutant NS3. (B) NS3 greatly accelerates RNA annealing without ATP hydrolysis. Representative gels of RNA annealing time courses in the absence and presence of mutated NS3Amut and ATP (as indicated on top of each gel). The annealing reactions were carried out as described in Fig. 4B. Underneath, a graph showing representative time courses of RNA annealing obtained by quantification of bands in the gel at the indicated conditions. The continuous lines represent the best fits to the differential equations for a one-step bimolecular reaction (see Materials and Methods).

Some but not all RNA helicases have both RNA unwinding and RNA annealing activities [56]–[59]. In this regard, it has been recently reported that NS3 from HCV displays RNA annealing activity, which is ATP dependent [60]. Our results using DENV NS3 suggested that the RNA annealing activity was independent of ATP. To further examine this activity, we designed a mutant NS3-hel carrying a two amino acid substitution (D284A-E285A) in the conserved motif II, which corresponds to the Mg^2+^ co-factor binding loop. This mutation was previously described to impair ATP hydrolysis [28]. Expression and purification of this protein (NS3Amut) was identical to that for the WT protein. Reactions to evaluate ATPase and helicase activities indicated the lack of these enzymatic functions in the mutated NS3 (Fig. 5A).

To determine whether the NS3Amut retained RNA annealing activity, the structured 58 nucleotide-long RNA was incubated with the complementary Cy5 labeled probe in the presence and absence of protein. In the absence of protein only a small fraction of probe was annealed (Fig. 5B, left panel). Hybridization of the two complementary RNAs was highly accelerated by the presence of NS3Amut, confirming that RNA annealing activity of DENV NS3 is ATP independent (Fig. 5B, central panel). In addition, in the presence of ATP, the hybridization kinetics was indistinguishable from that observed in the absence of ATP (Fig. 5B, right panel). The initial velocities of RNA annealing observed in the presence of NS3Amut were 0.93±0.04 and 0.99±0.06 nM min^−1^, in the absence and presence of ATP, respectively. The lack of helicase activity in the mutant protein allows completion of the annealing reaction even in the presence of ATP. The results conclusively demonstrate that DENV NS3 contains ATP-dependent RNA helicase activity and ATP-independent RNA strand annealing activity. In addition, the data show that, in our experimental conditions using small RNA molecules, the helicase activity is not necessary in unfolding secondary structures of the RNA substrates during the annealing reaction.

**Figure 6:**
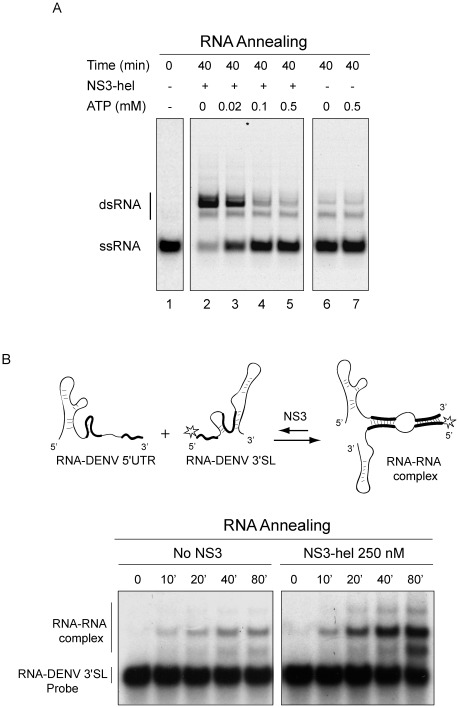
NS3 modulates viral and non-viral RNA structures. (A) Effect of ATP concentration on RNA unwinding/annealing activities of NS3-hel. Representative gels showing RNA annealing activity in the presence of different ATP concentration. A negative control at time 0 shows the mobility of the ssRNA probe (lane 1). A control without protein shows the spontaneous annealing (lanes 6 and 7). In the presence of NS3-hel at 40 min., the result of RNA annealing/unwinding is shown at different ATP concentrations, as indicated on top of the gel (lanes 2–5). (B) NS3-hel modulates biologically relevant RNA-RNA interactions between elements present at the ends of the DENV genome. On the top, a schematic representation of the viral 5′UTR and the 3′ stem-loop (3′SL) including the cyclization sequences (shown in bold). Hybridization of the complementary sequences results in an RNA-RNA complex. A representative gel showing time courses of RNA-RNA complex formation in the presence and absence of NS3-hel is shown. Mobilities of the 3′SL probe and the RNA-RNA complex are indicated on the left.

Our findings suggest that ATP availability modulates the RNA remodeling activity of DENV NS3. In this regard, a reaction initiated from single-stranded RNA substrates, including different concentrations of ATP, shows that the RNA annealing activity prevails at low ATP concentration, while when the ATP concentration increases the annealing and unwinding activities are observed (Fig. 6A).

We have recently provided experimental evidence that support the idea that DENV genome exists in at least two alternative conformations [9]. RNA structures present at the ends of the viral genome form hairpins by local interactions, or RNA duplexes by long range RNA-RNA interactions. Here, we tested the ability of NS3 to modulate this functionally relevant viral RNA structures. Hybridization of an RNA molecule containing the sequence of the viral 5′UTR (including the 5′-cyclization sequences) with a second radiolabeled RNA corresponding to the last 106 nucleotides of the viral genome was evaluated by mobility shift assay (Fig. 6B, top scheme). Spontaneous hybridization of the ends of the genome was very slow, unless molecules were heated and cooled slowly [6]. In the presence of NS3, a displacement of the equilibrium was observed towards the formation of the RNA-RNA complex, indicating that the annealing activity of NS3 is able to change viral RNA structures (Fig. 6B). We propose that the participation of NS3, modulating the folding of cis-acting RNA elements by the dual RNA unwinding/annealing activities, provides a new biological role for this viral protein.

## Discussion

Secondary and tertiary RNA structures present in viral genomes are crucial for viral replication. However, little is known about the processes that regulate their formation and dynamics. Here, we investigated the ability of the DENV NS3 protein not only to disrupt double-stranded RNA structures but also to facilitate hybridization of complementary RNA molecules. We found that NS3 greatly accelerates RNA annealing in the absence of ATP. The capacity of NS3 to enhance RNA annealing and unwinding reactions, and the possibility to differentially modulate these activities, provide new ideas of how viruses can regulate RNA conformations in different stages of the viral life cycle.

We first investigated a controversial issue regarding the requirement of the protease domain of NS3 for helicase activity. Extensive structural and functional studies have conclusively demonstrated a strong interdependence in the enzymatic activities of the protease and helicase domains of HCV NS3 protein [61]–[64]. In contrast, previous reports using flavivirus NS3 proteins have provided dissimilar results. It has been reported that DENV4 NS3 full-length protein displayed 10-fold higher ATP affinity than the isolated helicase domain [38]. In addition, for DENV2 NS3, the RNA unwinding activity was about 30-fold higher than that of the NS3-hel [35]. Also, a higher rate of RNA unwinding was reported for Kunjin virus (KUNV) full-length NS3 when compared to different variants of NS3-hel (169–619, 180–619 and 186–619) [37]. These observations originally suggested a regulatory role of the protease domain on flavivirus NS3 helicase activity. More recent studies using Murray Valley encephalitis virus (MVEV) NS3 indicated that the protease had little influence on the helicase activity and vice versa [26]. These findings were in agreement with structural data using the MVEV NS3, which indicated that the protein contained two structurally independent domains [26]. To investigate the influence of the protease domain on the activity of the DENV NS3 helicase domain, we performed a rigorous analysis using viral proteins with robust enzymatic activities (Fig. 1). Our studies indicate that the helicase domain, encompassing amino acids 171 to 618, has steady-state kinetic properties with *k*_cat_ and *K*_M_ constants for ATP hydrolysis similar to that observed for the full-length NS3. In addition, no significant differences between the two enzymes were observed in regard to RNA allosteric activation of ATP hydrolysis and RNA unwinding time courses (Fig. 1). It is possible that the discrepancies with previous reports were due to distinct N-terminal boundaries of the NS3-hel constructs or different protein preparations. In this regard, the functional domains required for NS3 protease and helicase mapped within a region of twenty amino acids (160 to 180 of NS3) [27]. This linker domain is flexible and mutations that alter flexibility have been shown to alter protein functionality [65]. Together, our results indicate that the ATPase and helicase activities, as well as the allosteric RNA stimulatory effect on the ATPase activity of DENV NS3, are not significantly affected by the protease domain (Fig. 1 and 2).

Previous structural and biochemical studies have shown that many RNA helicases have a distinct preference for NTPs. The Q-motif, a common theme presents in many helicases, conferes specificity for ATP, while helicases lacking this motif (like NS3) are able to utilize diverse NTPs [66]. In agreement with these previous reports, we established that several hydrolyzable nucleotides, and not only ATP, were substrates for the RNA unwinding activity of NS3. Canonical NTPs as well as dATP and ddATP functioned efficiently as energy sources to fuel the unwinding reaction (Fig. 2).This broad substrate specificity of NS3 can be used to design nucleotide derivatives as inhibitors of the viral enzyme. In addition, to study the ability of NS3 to hydrolyze NTP analogues is important in the context of the efficiency of antiviral inhibitors acting as chain terminators of RNA synthesis. In this regard, it has been previously reported that HCV NS3 is capable of hydrolysing ribavirin, used as RNA polymerase inhibitor, decreasing its antiviral activity [67].

Using RNA molecules with complementary regions involved in local interactions, and employing conditions in which spontaneous hybridization was unfavourable, high strand annealing activity of the DENV NS3 protein was observed (Fig. 4). Acceleration of RNA annealing appears to take place through a mechanism that is different from the reversal of the unwinding activity, since RNA annealing did not require ATP. This observation was unexpected because in a recent report an RNA annealing activity of HCV NS3 has been described to require ATP [59]. To confirm our observations, a mutated DENV NS3-hel protein was designed including two-amino acid substitution that impaired NTPase activity. This enzyme lacked unwinding activity but retained full RNA annealing capacity (Fig. 5B). These results indicate that the helicase activity of dengue virus NS3 was not required to unfold structures present in the RNA molecules used in this study under our experimental conditions (Fig. 4A, Fig. 5B, and Fig. 6B). It is possible that binding of NS3 to the RNA is sufficient to promote annealing. There are indications that NS3 could oligomerize under certain conditions. In this regard, NS3-NS3 interaction could bring different RNA molecules into proximity, accelerating RNA annealing. In addition, protein binding could result in conformational changes in the RNA. This could stabilize extended RNA structures in which the nucleotides are more available for interaction. The binding of NS3 could also cause a shielding of the charges of the phosphate backbone, which could contribute to RNA annealing activity by reducing intermolecular repulsion. Nevertheless, we cannot rule out that annealing of highly structured RNA molecules requires a helicase-dependent unwinding activity. Further studies will be necessary, using more complex RNA substrates, to determine the interplay between helicase and RNA annealing activities of NS3.

RNA annealing is not considered a general biochemical characteristic of RNA helicases. However, a growing number of proteins with RNA remodelling activities are being reported, including enzymes that accelerate RNA strand annealing [4]. The requirements for RNA annealing/unwinding activities appear to be different in different proteins. In some cases, both reactions require ATP. For instance the cyanobacterial CrhR displays ATP-dependent RNA annealing activity [57], while the yeast nuclear DEAD-box RNA helicases, p68 and p72, are ATP-independent annealers [58], [68]. Interestingly, the human Ddx42p and the Saccharomyces cervisae DED1 proteins have been reported to have strong RNA annealing activities that are modulated by ADP [58], [68].

It is still unclear how viral RNA helicases participate in viral replication. Moreover, the physiological significance of combined RNA unwinding and RNA annealing activities in a viral protein has not been investigated. We have recently provided a model in which the DENV genome is a dynamic RNA molecule and at least two mutually exclusive conformations are crucial for infectivity [9]. In this study, we show that NS3 is able to enhance hybridization of RNA structures that resemble the ends of the viral genome (Fig. 6). These conformational changes were previously shown to be necessary for viral RNA synthesis [6]–[17]. We propose that the availability of ATP in different compartments of the infected cell or in different stages of the viral life cycle could change, regulating NS3 unwinding or annealing activities. In this regard, significant ATP concentration changes in different cellular compartments have been recently detected in living cells by a novel real time monitoring system [69].

Although we found that the protease domain does not modulate the helicase activity of DENV NS3 in vitro, it is likely that in the infected cell the two enzymatic activities (or other functions of NS3) are necessary in the same polypeptide. Viral polyprotein processing and RNA replication occur in distinct compartments known as convoluted membranes and vesicle packets, respectively (for review see [70]). It is possible that the membrane association of NS3 through NS2B allows localization of NS3-hel in a specific subcellular compartment. For different flaviviruses a role of the NS3 helicase domain in viral assembly has been reported [71], [72]. However, the mechanism by which NS3 participates in this viral process is still unclear. It is possible that viral RNA annealing and unwinding are necessary for genome recruitment during particle morphogenesis or for mobilizing the genome between different compartments of the infected cell. In addition, an important role of NS3 in counteracting the host antiviral response has been recently described [73]. For this function, the NS2B-NS3 protease domain was required. Because the recognition of specific properties of the viral RNA by pattern recognition receptors triggers type I interferon production [74], [75] we can speculate that regulation of RNA structures by NS3 could also play a role in modulating the antiviral host response.

We believe that finding a novel enzymatic activity associated to NS3 not only helps understanding the role of this essential viral protein, but also provides new opportunities for antiviral intervention.

## Materials and Methods

### Cloning and site-directed mutagenesis of DENV2 NS3

Constructs encoding NS3 proteins were derived from the cDNA of an infectious clone of DENV2 strain 16681 (GenBank accession number U87411) [76]. Sequences of PCR primers are presented in Table 1. Full-length NS3 protein fused to a central region of NS2B was designed as follows: the DNA fragment corresponding to residues 49 to 95 of the NS2B protein was amplified by PCR using forward primer AVG492 (which incorporates a *BamHI* restriction site at the 5′ terminus) and reverse primer AVG119 (which incorporates the nucleotide sequence encoding the GGGGSGGGG linker sequence at the 3′ terminus). The DNA fragment corresponding to amino acid residues 1 to 618 of NS3 protein was amplified by PCR using the forward primer AVG120 (which incorporates the nucleotide sequence encoding the GGGGSGGGG linker sequence at the 5′ terminus) and the reverse primer AVG16 (which incorporates a stop codon and a *HindIII* restriction site at the 3′ terminus). From these two PCR products, an overlapping PCR was carried out using primers AVG492 and AVG16 and then cloned into a pET-28a vector (Novagen) between *BamHI* and *HindIII* restriction sites to generate plasmid pET-CF47-NS3-FL. This plasmid encodes for a polypeptide consisting of an N-terminal hexahistidine tag (derived from the vector) followed by the central hydrophylic region of NS2B linked by a GGGGSGGGG sequence to the full-length NS3 protein. Recombinant protein preparations from this recombinant plasmid resulted in NS3 degradation due to the presence of the active two-component NS2B-NS3 protease domain. To avoid this problem, the active site of the protease was mutated (histidine residue at position 51 of the catalytic triad was replaced for alanine). For this mutation, plasmid pET-CF47-NS3-FL was used as template and primers AVG118, AVG541, AVG492 and AVG16 were used to generate plasmid pET-CF47-NS3-FL-H51A by overlapping PCR. His51 to Ala replacement greatly decreased auto-proteolysis.

**Table 1:** Sequence of oligonucleotides used as primers for PCR.

Name	Construct	Sequence (5′ to 3′)
AVG16	pET-CF47-NS3-FL	TTAGCGAAGCTTACTACTTTCTTCCGGCTGCAAATTC
AVG119	pET-CF47-NS3-FL	CCCGCCTCCACCACTACCTCCGCCCCCCAGTGTTTGTTCTTCCTC
AVG120	pET-CF47-NS3-FL	GGGGGCGGAGGTAGTGGTGGAGGCGGGGCCGGAGTATTGTGGGATG
AVG492	pET-CF47-NS3-FL	TAATGGATCCAGCATTGAAGACAACCCAGAGATCGAAG
AVG118	pET-CF47-NS3-FL-H51A	GGATCCGCCGATTTGGAACTG
AVG541	pET-CF47-NS3-FL-H51A	CCTTTATGCATTAGAACAGCGCCTCGTGTGACAGCCCACATTGTATGGAATG
AVG943	pET-NS3-hel	ATCCATGGGCCATATGAGCATTGAAGACAACCCA
AVG1262	pET-NS3-hel-D284E285AA	ATTATCATGGCGGCCGCCCATTTCAC
AVG1263	pET-NS3-hel-D284E285AA	GAAATGGGCGGCCGCCATGATAATCA
AVG689	5′DV-159 DNA template	TCGTTAATACGACTCACTATAGGTTGTTAGTC
AVG130	5′DV-159 DNA template	GTTTCTCTCGCGTTTCAGCATATTG
AVG11	3′SL DNA template	TAATACGACTCACTATAGGCAGCATATTGACGCTGGGAAAG
AVG5	3′SL DNA template	AGAACCTGTTGATTCAACAGCAC

The DNA fragment encoding for DENV2 NS3 helicase domain (residues 171 to 618) was obtained by PCR from plasmid pET-CF47-NS3-FL using forward primer AVG943 (which incorporates an *NdeI* restriction site at the 5′ terminus) and reverse primer AVG16. The PCR product was digested with *NdeI* and *BamHI* and cloned into a modified pET-28a plasmid using T4 DNA ligase to generate plasmid pET-NS3-hel. This plasmid encodes for a polypeptide consisting of an N-terminal hexahistidine tag followed by the polypeptide corresponding to the helicase domain of NS3 protein (residues 171 to 618). In order to generate an ATPase-defective variant of NS3-hel, Asp284 and Glu285 residues were replaced for alanine by site-directed mutagenesis. Plasmid pET-NS3-hel-D284E285-AA was generated by overlapping PCR using plasmid pET-NS3-hel as template and primers AVG943, AVG1263, AVG1262 and AVG16.

*Pfx* DNA polymerase was purchased from Invitrogen. Restriction enzymes, T4 DNA ligase and calf-intestinal alkaline phosphatase were obteined from New England Biolabs. DNA sequences were confirmed by automatic DNA sequencing using a 3130 Genetic Analyzer (Applied Biosystems).

### Protein purification

Competent *E. coli* BL21 (DE3) Rosetta pLac cells were transformed with expression plasmids. Transformed cells were grown at 37°C in LB medium containing kanamycin (50 mg/L) and chloramphenicol (34 mg/L) to an optical density at 600 nm of 0.6–0.8. Then, 0.5 mM IPTG was added in order to induce protein expression. After 4 hours at 28°C, cells were harvested by centrifugation and stored at −80°C. Cells were suspended in 3 ml of buffer A (50 mM HEPES-KOH, pH 7.0, 5% glycerol, 500 mM KCl, 0.1 mM DTT, 20 mM imidazole) supplemented with 2 mM DTT, 2 mM MgCl_2_, 2 mg/ml DNAse I, 10 units protease inhibitor cocktail (Roche), and then lysed by a French press (SLM Instruments Inc., Urbana, IL) and clarified by centrifugation (20 minutes at 20,000×g at 4°C). The cell extract supernatant was applied to a HisTrap HP column (GE Healthcare, Uppsala, Sweden) previously equilibrated in buffer A and eluted in a gradient with buffer B (buffer A supplemented with 500 mM imidazole chloride, pH 7.0) using an AKTA FPLC (GE Healthcare). Fractions containing the protein of interest were pooled, dialyzed exhaustively against buffer D (50 mM HEPES-KOH, pH 7.0, 5% glycerol, 200 mM KCl, 0.2 mM DTT) at 4°C using a 14 kDa cut-off dialysis tube (Spectrum Laboratories Inc.).The sample was then concentrated to 10 mg/ml using an Amicon Ultra centrifugal filter unit (Millipore Corporation). Particulate material was removed by centrifugation at 16,000×g for 30 minutes at 4°C. The supernatant fraction was stored at −70°C.

### RNA substrates

Synthetic RNA oligomers (up to 30-mer) were purchased from Integrated DNA technologies Inc. Longer RNAs (5′DV-159, 3′SL, and RNA58) were obtained by in vitro transcription using T7 RNA polymerase (Ambion) and treated with RNase-free DNase I to remove DNA templates. These RNAs were purified using illustra MicroSpin G-50 Columns (GE Healthcare) to remove free nucleotides, and then quantified spectrophotometrically. Their integrities were verified by electrophoresis on agarose gels. All numbers given below in parentheses refer to nucleotide positions of a dengue virus type 2 strain 16681 infectious cDNA clone (GenBank accession number U87411). The sequences corresponding to 5′DV-159 (nucleotides 1 to 159) and 3′SL (nucleotides 10617 to 10723) were amplified by PCR using primers AVG689 and AVG130, and AVG11 and AVG5, respectively, from a DENV2 cDNA clone [6] with a forward primer carrying the T7 RNA polymerase promoter. 3′SL RNA, previously treated with calf intestinal phosphatase (New England Biolabs), was radiolabeled at the 5′-end using ^32^P-γ-ATP (Perkin Elmer) and T4 polynucleotide kinase (New England Biolabs).

RNA58 (5′-GGAUCGCAGCUGACUGCGAUCCGACUGUCCUGCAUGAUGCAGGACAGUAACAGGUUCU-3′) was designed to fold in two hairpins including a sequence that is complementary to the Cy5-RNA30 probe (underlined in the sequence).

### ATPase assay

ATP hydrolysis was determined by using EnzCheck Phosphate Assay Kit (Molecular Probes, Invitrogen Inc.). Purified recombinant NS3 variants at a concentration of 40 nM were preincubated for 10 minutes at 25°C in 150 µl buffer N (25 mM HEPES-KOH, pH 7.0, 20 mM KCl, 5% glycerol, 1.5 mM MgCl_2_, 2 mM dithiothreitol, 0.05% CHAPS, 0.01 mM EDTA, purine nucleoside phosphorilase, 0.2 mM MESG). The reaction was initiated by adding ATP (including an equimolar concentration of MgCl_2_) in 150 µl buffer N and carried out in a spectrophotometer microcuvette. The initial velocities of ATP hydrolysis were calculated as previously described [66]. The results were fitted to the Michaelis-Menten equation by nonlinear regression to determine *k*_cat_ and *K*_M_ constants. RNA-stimulated ATPase activity was determined as described above, including different concentrations of a synthetic RNA (20-mer) and 2 mM ATP-MgCl_2_.

### RNA helicase activity assay

In order to generate substrates for helicase assays, two synthetic oligonucleotides (Integrated DNA Technology Inc., IDT) were annealed by heating at 90°C and cooled slowly to room temperature. The duplex RNA substrate contained an RNA strand of 30 nucleotides in length (5′-CAUCAUGCAGGACAGUCGGAUCGCAGUCAG-3′) annealed to a shorter RNA strand of 15 nucleotides (5′-CUGUCCUGCAUGAUG-3′). When the 15-nucleotide long molecule was radio-labeled, a polynucleotide kinase (New England Biolabs Inc) and ^32^P-gamma-ATP (Perkin-Elmer Inc) were used. The 5′Cy5 labeled 30-mer RNA was purchased from IDT.

RNA unwinding reactions were performed at 30 or 37°C in a final volume of 20 µl. RNA substrate (5 nM) and NS3 (250 nM) were preincubated for 10 minutes in buffer R (25 mM HEPES-KOH, pH 7.0, 40 mM KCl, 5% glycerol, 4 units of RNAse inhibitor, 0.5 mM MgCl_2_, 2 mM dithiothreitol, 0.05% CHAPS, 0.01 mM EDTA). Unwinding reactions were initiated by adding ATP in the indicated concentrations. Simultaneously the RNA trap (100 nM of unlabeled oligonucleotide) was added in order to prevent re-annealing of the probe. Reactions were terminated at different incubation times by adding 2 µl of buffer S (1.2 mg/ml proteinase K, 5.0 mg/ml heparin, 1.0% sodium dodecyl sulphate, 10% glycerol) in order to inactivate the enzyme and to dissociate NS3 from the RNA probe, preventing formation of RNP complexes. Reaction mixtures were resolved on non-denaturing 9% polyacrylamide gels in TBE buffer (20 mM Tris-HCl, 20 mM boric acid, 0.5 mM EDTA, pH 8.0) supplemented with 5% glycerol using Mini-PROTEAN electrophoresis system (Bio-Rad Laboratories Inc., USA). The fluorescent signal from Cy5-labeled probes was detected by scanning the gels with a Storm 865 device (GE Healthcare). The radioisotopic probes were detected by using autoradiographic plates (Kodak Inc.). The signal was quantified with ImageJ software (NIH). The ATP regeneration system included creatine phosphokinase (32 µg/ml) and creatine phosphate (16 mM) (Roche Diagnostics Corporation, USA).

### RNA strand annealing activity assay

RNA strand annealing activity of NS3 was assayed in a 20 µl volume containing the single-stranded RNA probe (5 nM or at the concentration indicated in each case) and the complementary RNA strand (20 nM or at the indicated concentration). Reaction mixtures were incubated with NS3 (250 nM) and Mg-ATP at different concentration as indicated in each case. Annealing reactions were initiated by mixing aliquots of the two RNA strands in buffer R. Reactions were terminated by cooling the sample on ice to detain the annealing process. Simultaneously, 2 µl of buffer S were added to inactivate the enzyme and to prevent formation of RNP complexes. Single-stranded and duplex RNAs were separated and detected as described above for the helicase activity assay.

### Data Analysis

The amount of ssRNA and dsRNA were determined from the intensity of the respective bands in the gels measured by ImageJ 1.45 software (National Institutes of Health, USA). Observed rate constants and amplitudes (*k* ^unw^_obs_ and *A*, respectively) for unwinding reactions were determined by fitting time courses to a monoexponential function of time:
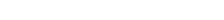
Initial velocity for unwinding reactions were obtained as follows,
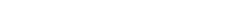
Time course for strand annealing were fitted by numerical integration of the differential equations for a one-step bimolecular reaction (ssRNA_1_+ssRNA_2_ = dsRNA); and the initial velocities were obtained as, v_i_ = (d [dsRNA]/dt)_t = 0_ from the slopes of the fitting curves at their zero time.

Equations and numerical solution of the kinetic models were adjusted to the experimental data by non-linear regression analysis using free software COPASI. This program provides not only the best fitting values of the parameters but also their standard errors [77].
